# Intestinal Flora-Derived Kynurenic Acid Protects Against Intestinal Damage Caused by *Candida albicans* Infection *via* Activation of Aryl Hydrocarbon Receptor

**DOI:** 10.3389/fmicb.2022.934786

**Published:** 2022-07-18

**Authors:** Zetian Wang, Liping Yin, Yue Qi, Jiali Zhang, Haiyan Zhu, Jianguo Tang

**Affiliations:** ^1^Department of Trauma-Emergency & Critical Care Medicine, Shanghai Fifth People’s Hospital, Fudan University, Shanghai, China; ^2^Department of Central Laboratory, Shanghai Fifth People’s Hospital, Fudan University, Shanghai, China; ^3^Department of Biological Medicines & Shanghai Engineering Research Center of Immunotherapeutics, Fudan University School of Pharmacy, Shanghai, China

**Keywords:** intestinal flora, invasive *C. albicans* infections, aryl hydrocarbon receptor, kynurenic acid, intestinal barrier function

## Abstract

Colonization of the intestinal tract by *Candida albicans* (*C. albicans*) can lead to invasive candidiasis. Therefore, a functional intestinal epithelial barrier is critical for protecting against invasive *C. albicans* infections. We collected fecal samples from patients with *Candida albicans* bloodstream infection and healthy people. Through intestinal flora 16sRNA sequencing and intestinal metabolomic analysis, we found that *C. albicans* infection resulted in a significant decrease in the expression of the metabolite kynurenic acid (KynA). We used a repeated *C. albicans* intestinal infection mouse model, established following intake of 3% dextran sulfate sodium salt (DSS) for 9 days, and found that KynA, a tryptophan metabolite, inhibited inflammation, promoted expression of intestinal tight junction proteins, and protected from intestinal barrier damage caused by invasive Candida infections. We also demonstrated that KynA activated aryl hydrocarbon receptor (AHR) repressor *in vivo* and *in vitro*. Using Caco-2 cells co-cultured with *C. albicans*, we showed that KynA activated AHR, inhibited the myosin light chain kinase-phospho-myosin light chain (MLCK-pMLC) signaling pathway, and promoted tristetraprolin (TTP) expression to alleviate intestinal inflammation. Our findings suggest that the metabolite KynA which is differently expressed in patients with C. *albicans* infection and has a protective effect on the intestinal epithelium, *via* activating AHR, could be explored to provide new potential therapeutic strategies for invasive C. *albicans* infections.

## Introduction

As a severe systemic inflammatory response syndrome, sepsis is one of the leading causes of multiple organ dysfunction (Kuang et al., 2021). During the development of sepsis, the functions of intestinal barriers are altered. Impaired intestinal barriers allow for the invasion of intestinal bacteria and entry of endotoxins into the blood and lymph circulation, eventually causing a “second attack” and secondary pancreatic infection and sepsis (Li H. Y. et al., 2021). *Candida albicans* (*C. albicans*) is a member of the intestinal commensal microbiota that colonizes on the mucosal surfaces of the gastrointestinal tract (Jenull et al., 2021; Li H. Y. et al., 2021). This yeast can translocate into the bloodstream through impaired gut barriers in susceptible individuals, such as patients with sepsis, resulting in opportunistic infections (Hirao et al., 2014).

The intestinal microbiota is critical for human health. An accumulating body of evidence points out the key role of intestinal flora in maintaining intestinal homeostasis (He et al., 2020). Furthermore, numerous studies have shown that the intestinal flora can also regulate intestinal movement and secretion, decompose macromolecular complex polysaccharides in food, digest and absorb nutrients, maintain the integrity of the intestinal epithelial barrier, and promote and maintain the development and functions of the immune system (Dinan and Cryan, 2017; Li X. J. et al., 2021; Tian et al., 2021). Invasive *Candida* infections have been shown to alter the microecology of gut bacteria and aggravate intestinal damage. Studies using mice demonstrated that diallyl disulfide (DADS) can modulate gut microbiota and metabolites, as well as provide intestinal protection and alleviate *C. albicans* infections (Hu et al., 2021). The increase in intestinal microflora and their metabolites may represent potential strategies for the prevention and treatment of invasive *C. albicans* infections.

The aryl hydrocarbon receptor (AHR) resides in the cytosol and participates in multiple biological processes, such as cell proliferation, differentiation, and immune cell function (Parent et al., 2011; Liu et al., 2018). As a ligand-activated transcription factor, AHR exerts an anti-inflammatory effect on gut barrier damage (Liu et al., 2018). In the presence of ligands, such as 6-formylindolo(3,2-b)carbazole (FICZ), AHR translocates to the nucleus and dimerizes with the AHR nuclear translocator (ARNY) to initiate the transcription of target genes, including cytochrome P450 (CYP1A1), which contains functional AHR responsive elements (AhRES) (Liu et al., 2018; Gasaly et al., 2021). Although previous studies reported activation of AHR by MG132 to alleviate liver injury in *in vivo* and *in vitro* models of intestinal ischemia/reperfusion, the mechanisms underlying the effect of AHR activation on intestinal barrier damage following invasive *C. albicans* infection remain unknown (Arda-Pirincci and Bolkent, 2014).

Tristetraprolin (TTP) is an mRNA-binding and decaying protein that can control inflammation response through a decrease of TNF-αtranscription (Patil et al., 2008). Furthermore, the mitogen-activated protein kinase-2/phosphorylated mitogen-activated protein kinase-2 (MK2/p-MK2) pathway can regulate TTP stability, expression, and function (Wang W. et al., 2018). Recent research has shown that the MK2/p-MK2 signaling cascade regulates TTP-mediated mRNA stability of IL-6 and TNF-α (Sun et al., 2011). Another study suggested that AHR reduces inflammation in experimental colitis *via* the downregulation of the MK2/p-MK2/TTP pathway (Ghiboub et al., 2020).

The interaction between multiple tight junctions contributes to the integrity of the intestinal epithelial barrier (Serlin et al., 2015). Tight junction regulation is mediated by myosin light chain kinase (MLCK), which phosphorylates the myosin II regulatory light chain (MLC) (Meng et al., 2013). Myosin light chain kinase (MLCK) controls the permeability of the endothelial cell (IEC) barrier by directly phosphorylating the myosin light chain (MLC) (Cheng et al., 2015). It can induce the activation of the MLCK-pMLC phosphorylation signaling pathway under various pathological conditions [such as hypoxia, lipopolysaccharide (LPS) stimulation, burn injuries, and inflammatory bowel disease (IBD), among others], leading to a decrease in the expression of the tight junction protein Zonula occludens protein 1 (ZO-1), disruption of intestinal mucosal barrier continuous distribution, and increase in the permeability of the intestinal mucosal barrier and damage of intestinal mucosal barrier function (Song et al., 2019). AHR can affect the expression and location of tight junctions in models of intestinal obstruction by regulating the MLCK-pMLC signal pathway, thereby improving the dysfunction of the intestinal mucosal barrier (Yu et al., 2018). In this study, based on differential metabolite analysis in patients with *C. albicans* infection, we hypothesized that metabolites of intestinal flora could activate AHR to protect against intestinal damage induced by *C. albicans* infection.

## Materials and Methods

### Human Samples

On the day of admission, samples of human feces were collected from healthy subjects and from patients with candidemia (six cases per group) at Shanghai Fifth People’s Hospital, Shanghai, China. The study protocol was approved by the Human Research Ethics Committee of Shanghai Fifth People’s Hospital, School of Medicine, Shanghai, China (Reference No. 2019-118). Participants or their guardians provided informed consent. Samples were frozen and stored in aliquots at 80°C in polyethylene tubes until use. The diagnostic criteria of candidemia were based on the guidelines for the diagnosis and treatment of Candidiasis: the expert consensus issued by the Chinese Medical Association. These criteria were also in accordance with the European Society of Clinical Microbiology and Infectious Diseases (ESCMID)* guidelines for the diagnosis and management of Candida diseases 2012 and the Infectious Diseases Society of America (IDSA) Guidelines for the Management of Candidiasis: 2016 Update (Pappas et al., 2009). Exclusion criteria were as follows: (a) age < 18 years old, (b) pregnant women, (c) the blood culture was found to be contaminated or there was no bloodstream *Candida* infection, and (d) loss to follow-up.

### 16S rRNA Analysis of the Microbial Community

Fecal samples were collected from patients and healthy controls (six cases per group). Briefly, DNA was extracted from feces by means of a Standard DNA Extraction Kit (QIAGEN) and analyzed by agarose gel electrophoresis to assess DNA quality. The V3-V4 region of 16S rRNA genes was amplified and purified. The Illumina MiSeq platform was used to sequence the V3-V4 gene amplicons. Raw data were filtered, and clean tags were removed to obtain valid tags for preparing operational taxonomic units (OTUs), which were classified using the Vsearch software (version 2.4.2) with a sequence similarity threshold of 97%. Then, the pynast (v0.1) software was used to create a phylogeny based on OTU sequence comparisons. A rarefied OTU table was used to determine the diversity and composition of the intestinal microbiota. Alpha-diversity indices for fecal samples were calculated using a normalized OTU table and a uniform depth. Based on the Bray-Curtis algorithm and unweighted UniFrac distance, beta-diversity indexes were generated to determine whether there were significant differences in the gut microbiota between groups (Chen et al., 2016). Principal component analysis (PCA) was also used to determine whether such differences existed.

### Fecal Metabolome Analysis

Six fecal extract samples were prepared for each group by combining 100 mg of fecal samples with 500 mL of ice-cold water, vortexed, and centrifuged at 13,000 rpm for 15 min at 4°C. Then, the supernatant was filtered through a 0.22 μm microfilter and stored at −80°C for liquid chromatography–mass spectrometry (LC–MS) analysis. The quality control (QC) group was formed by pooling equal volumes of supernatant from each sample to assess if the system’s mass spectrum platform remained stable throughout the experiment. Metabolite profiles were analyzed using an AB TripleTOF 6600 mass spectrometer (AB Sciex, United States) with Essential Science Indicator (ESI) sources in both positive and negative ion scan modes. Regarding MS TOF parameters, the fragmentor was set to 140 V and the skimmer to 65 V. All reagents used in this study were of high-performance liquid chromatography (HPLC) grade. The LC–MS data from fecal pellets were processed using Progenesis QI software (Waters Corporation, Milford, United States), and the metabolites were processed using the Progenesis QI Data Processing software. The ropls package in R was used to visualize the normalized data using principal component analysis (PCA) and orthogonal partial least squares-discriminant (OPLS-DA) analysis. With a 95% confidence interval threshold, the ellipses in PCA and OPLS-DA plots were used to characterize metabolic perturbation among groups in a Hotelling’s T2 region.

### *Candida albicans* Culture

*Candida albicans* (strain SC5314) was obtained from the China General Microbiological Culture Collection Center, Shanghai, China (CGMCC), and grown in a liquid medium containing yeast extract peptone dextrose (YEPD). Then, a single colony was streaked on a YEPD agar plate, incubated for 25 h at 35^°^C, and reidentified by mass spectrometry (Shanghai Fifth People’s Hospital, Fudan University, China). For preparation, inoculum containing 1.0 × 10^6^ cells of *C. albicans* clone was suspended in 0.3 mL of phosphate-buffered saline (PBS, pH 7.4).

### Mouse Experiments

Six to eight week-old male and female C57BL/6 mice weighing 20–23 g were acquired from East China Normal University’s Animal Center (Shanghai, China). All mice were kept in plastic boxes and fed food and water on a daily basis at 20–22^°^C with a 12-h light/dark cycle. Prior to experimentation, mice were left to acclimatize for 1 week. All animal experiments were authorized by the East China Normal University’s Experimental Animal Ethical Review Committee research (Shanghai, China). Mice were randomly divided into three groups. In the control group (*n* = 15), mice drank only sterile water without 3% dextran sulfate (DSS), and then were gavaged with 0.2 mL sterile autoclaved phosphate-buffered saline (PBS) every three days. In the CA group (*n* = 15), mice drank sterile water containing 3% DSS every day, and to induce intestinal mucosal destruction, the intestines of the mice were colonized with Candida albicans (1 × 10^6^ CFU, 0.2 mL) by oral gavage every 3 days, and mouse feces samples were collected for detecting the load of *Candida albicans*. In the CA+KynA group (*n* = 15), mice were treated as in the CA group but on the third day, KynA, at a dose of 10 mg/kg (0.2 mL), was administered at 6 h after intragastric administration of *C. albicans*.

**FIGURE 1:**
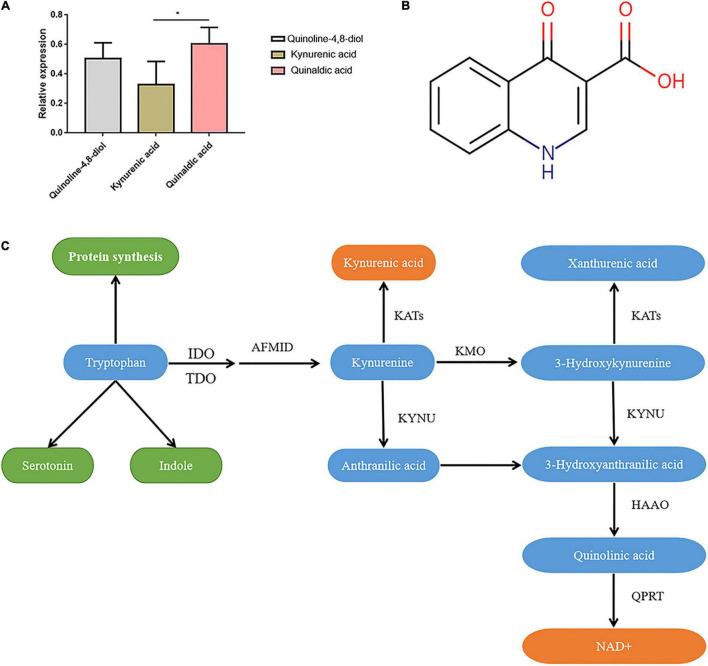
Characteristics of kynurenic acid. **(A)** The relative expression of Kynurenic acid in the peripheral blood of patients with invasive *C. albican* infection is significantly lower than the content of other metabolites (*P <* 0.05). **(B)** Kynurenic acid (HMDB0000715) is a kind of phosphatidic acid molecule with the molecular formula of C_10_H_7_NO_3_ and a molecular weight of 189.1675 Da. **(C)** The kynurenine pathway is the primary route for tryptophan metabolism, with one of the biologically active metabolites being kynurenic acid (KynA). T, The *Candida albicans* infection group; C, The healthy control group.

Mice were sacrificed on 4, 7, and 10 days. About 0.1 g of mouse excrements from the CA group (*n* = 5) and the CA+KynA group (*n* = 5) were collected in sterile tubes and diluted 10-fold with PBS. Mice were sacrificed 10 days after treatment with KynA, and the desired organs including the liver, spleen, and the kidneys were collected, weighed, and transferred to 1.5 mL sterilized EP tubes. A schematic diagram of the experimental design is shown in Figure 1A.

The suspension containing the feces and tissues was processed for gradient elution. About 100 μL of the suspension was added to 1 mL of sterile PBS solution and mixed well. Then, 10 μL of this solution was dissolved in 1 mL of sterile PBS solution, and coated on the culture medium. Colonies were counted after 3 days of incubation in an incubator at 37^°^C.

### Histomorphological Analysis

Colonic tissue samples from mice from all three groups were fixed in 4% neutral formalin, dehydrated with escalating concentrations of ethanol, and embedded in paraffin. Paraffin blocks were cut into 5-μm thick slices which were mounted on slides, cleaned, hydrated, and stained with hematoxylin and eosin (H&E). Two expert pathologists examined all specimens and were blind to the experimental group. Histologic alterations were evaluated using a modified grading system according to the amount of tissue damage. To establish a histopathological score, the following semi-quantitative parameters were used: (i) epithelial impairment, (ii) goblet cell decrease, (iii) inflammatory cell infiltration, and (iv) submucosal stiffness. The scores used were as follows: Score (i) included 0: normal, 2: distorted morphology of epithelial cells in one-third of total area, 4: distorted morphology of epithelial cells in more than one-third of total area and/or minor erosions, 6: occurrence of ulcers in 10% of ulcerated areas 8: 10–20% of ulcerated areas; and 10: > 20% of ulcerated regions). Scores (ii–iv) included 0: normal, 1: mild, 2: moderate, and 3: severe. The lowest possible score was 0 and the highest was 19.

### Western Blot Analysis

The cells and mouse intestinal tissues were lysed using an appropriate lysis buffer, and protein levels were determined using a BCA kit (Beyotime, China). Proteins were separated using SDS/PAGE in a Bio-Rad Mini-PROTEAN device before transfer to PVDF membranes (Bio-Rad, Marnes-la-Coquette, France). Membranes were then blocked for 1 h at room temperature with 5% non-fat milk (w/v), followed by overnight incubation with primary antibodies at 4°C. The following antibodies were used: anti-Occludin antibody (1:1,000, 13409-1-AP. Proteintech, United States); anti-AHR antibody (1:1,000, 67785-1-Ig; Proteintech, United States); anti-ZO-1 antibody (1:1,000, 21773-1-AP, Proteintech, United States); anti-GAPDH antibody (1:1,000, 60004-1-Ig, Proteintech, United States); anti-MK2 antibody (1:1,000, 13949-1-AP, Proteintech, United States); anti-CYP1A1 antibody (1:1,000, 13241-1-AP, Proteintech, United States); anti-TTP antibody (1:1,000, 12737-1-AP; Proteintech, United States); anti-MLCK antibody (1:1,000, 21642-1-AP; Proteintech, United States); and anti-pMLC antibody (1:2,000, CST-3671; Cell Signaling Technology, United States). The secondary antibodies used were as follows: HRP-conjugated goat anti-mouse IgG (H+L) (#115-035-003) and HRP-conjugated goat anti-rabbit IgG (H+L;#111-035-003), purchased from Jackson ImmunoResearch, United States. Protein expression was normalized to GAPDH, and densitometry of Western blot bandings was evaluated with Image J (Version 1.50i; National institutes of Health, Bethesda, MD, United States).

### Immunohistochemistry Staining

The tissues were fixed with 4% paraformaldehyde at 4°Covernight, embedded in paraffin, and sliced into 5-μm sections. Sections were dehydrated in an ethanol gradient at room temperature for 5 min, and treated with 0.3% hydrogen peroxide in methanol at room temperature for 20 min. Sections were then incubated in citrate buffer (pH 6.0) and microwaved for 20 min for antigen retrieval. Sections were incubated at 4°C overnight with an anti-AHR antibody (1:200, 67785-1-Ig; Proteintech, United States) and then blocked with 5% bovine serum albumin (9048-46-8, Merck) at room temperature for 20 min. For incubation with the secondary antibodies, sections were then incubated at 37°C for 20 min with biotinylated goat anti-mouse and rabbit secondary antibodies (Sa1020, ready to use) and avidin-biotin complex (Sa1020, ready to use), purchased from Wuhan Boster Biological technology ltd., China. Peroxidase activity was determined by staining with diaminobenzidine (0.5 mg/mL) at room temperature for 20 s. Histological evaluation was performed under a light microscope (magnification, x400; Nikon Corporation), after counterstaining with hematoxylin (1 g/L) at room temperature for 1 min.

### Enzyme-Linked Immunosorbent Assays

Five samples of peripheral blood were collected from each group and centrifuged at 3,000 rpm at 4°C for 10 min for serum collection. Serum was stored at -80 °C until use. Murine ELISA kits (DEIA1348, Creative Diagnostics, United States; DEIA-BJ2494, Creative Diagnostics, United States; Creative Diagnostics, United States) were used to measure the levels of IL-6, IFN, and D-lactic acid.

### Cell Culture

Human colorectal adenocarcinomas (Caco-2) cells obtained from the American Type Culture Collection (Invitrogen, Manassas, VA, United States) were cultured in Eagle’s Minimum Essential Medium supplemented with 10% heat-inactivated fetal bovine serum (Gemini Bioproducts, Calabasas, CA, United States) and 1% non-essential amino acids. Caco-2 cells were seeded on six-well plates at a density of 1 × 10^6^ cells/well. Once the monolayers reached 70–80% confluence, they were cultured with serum-free MEM basic media overnight and then co-cultured with 1 × 10^5^ CFU/mL concentration of *C. albicans* for 24 h, with or without 10 μM KynA (Selleck, United States), 100 nM FICZ (CAS No:172922-91-7, MeChemExpress, United States), and 10 μM CH223191 (CAS No:301326-22-7, MedChemExpress, United States), to examine the expression of AHR, CYP1A1, MLCK-pMLC, MK2-p-MK2, ZO-1, and occludin proteins.

### Statistical Analysis

Data were expressed as mean and standard deviation (SD). Group differences were assessed using a one-way analysis of variance with the Student–Newman–Keuls test and the SPSS statistical software package (version 13.0; SPSS, Inc., Chicago, IL, United States). A statistically significant difference was defined as *P* < 0.05. Survival statistics were performed using Kaplan–Meier curve and log-rank test.

## Results

### Invasive *Candida albicans* Infection Disrupts Fecal Microbiota Diversity and Metabolites in Humans

**FIGURE 2:**
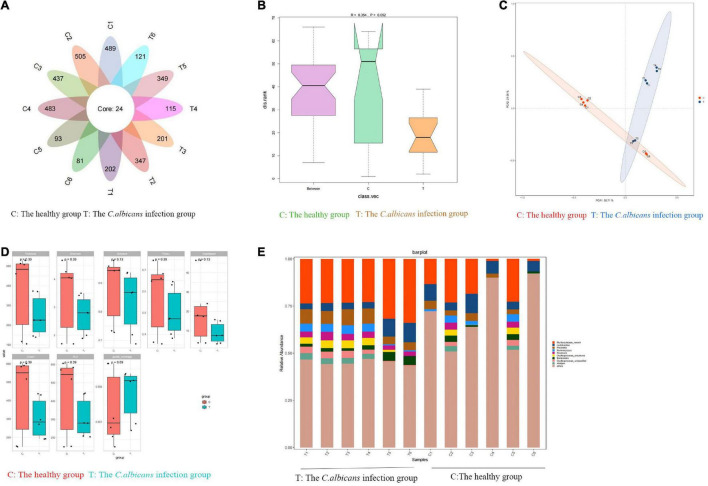
Invasive *C. albicans* infection disrupts fecal microbiota in humans. Human fecal samples were collected from healthy people (*n* = 6) and patients with candidemia (*n* = 6). 16S rRNA gene amplicon sequencing and metabolomic analysis were done to investigate the bacterial composition and intestinal metabolites. **(A)** The petal diagram of OTU distribution. **(B)** Analysis of similarities (ANOSIM). **(C)** The PCA analysis of OTU abundance. **(D)** Alpha-diversity analysis. **(E)** At the genus level, invasive C. albicans infection disrupts fecal microbiota diversity. T, The *Candida albicans* infection group; C, The healthy control group.

Fecal samples were collected from patients with candidemia (*n* = 6) and healthy subjects (*n* = 6) aged 55–65 years. To investigate the bacterial composition and intestinal metabolites, we used 16S rRNA gene amplicon sequencing and metabolomic analysis. A total of 3,423 OTUs were obtained from 12 samples. As shown in Figure 2A, in a petal diagram of OTU distribution, the number 24 in the core represents the number of OTUs shared by the two groups. According to the analysis of similarities (ANOSIM), there was a significant difference between the two groups of sampling units (Figure 2B). PCA analysis of OTU abundance in each group showed significant differences between the two groups (Figure 2C). Alpha-diversity analysis was used to estimate the microbial diversity of each individual sample. There was a significant difference in Shannon, Simpson, and Invsimpson in the candidemia group compared to the control group (*P <* 0.05). As shown in Figure 2D, invasive *C. albicans* infection decreases the diversity of flora. As expected, cluster analysis of species abundance based on the genus levels showed significant changes in the composition of gut microbiota between groups (Figure 2E). At the genus level, the relative abundances of *Lachnoclostridium*, *Bacteroides*, and *Lactococcus* were decreased in the candidemia group, whereas the relative abundances of *Bacillus, Enterobacter*, and *Caulobacter* were increased (*P <* 0.05).

**FIGURE 3:**
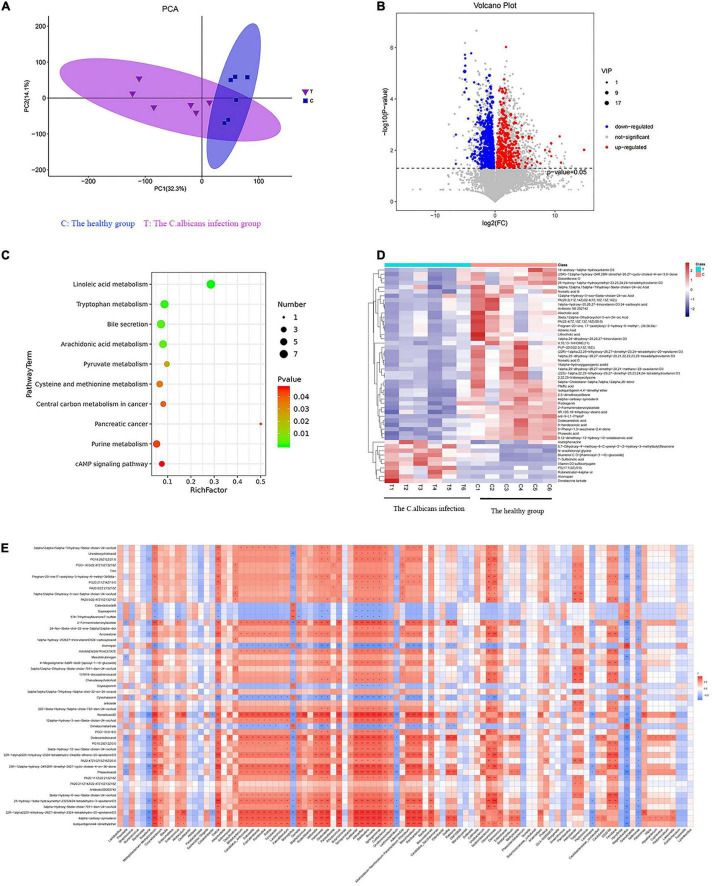
Invasive *C. albicans* infection disrupts fecal metabolites in humans. **(A)** The PCA scatter plots of metabolomic analysis. **(B)** The volcano plot of differentially expressed metabolites. **(C)** The KEGG pathway enrichment analysis. **(D)** Heatmap of the top 50 differentially expressed metabolites. **(E)** Spearman correlation analysis. T, The *Candida albicans* infection group; C, The healthy control group.

The metabolic changes are closely related to alterations in the gut microbiota, which are also regarded as a key feature of intestinal inflammation. We used LC–MS to identify differentially expressed metabolites and key metabolic pathways in the two groups. A total of 962 metabolites were identified among the 12 fecal samples. The PCA scatter plots revealed clustered QC samples, indicating the high quality of metabolomic analysis (Figure 3A). To identify key metabolites, metabolites were visualized on a heatmap. Nineteen metabolic pathways were found to differ significantly between the two groups. We identified differentially expressed metabolites with statistical significance between groups by volcano plot filtering (Figure 3B). KEGG pathway enrichment analysis of differentially expressed metabolites using the Fisher precise test revealed significant changes in important pathways, such as linoleic acid metabolism, tryptophan metabolism, bile secretion, and arachidonic acid metabolism, between the groups (Figure 3C). When the top 50 metabolites were visualized in a heatmap, differential metabolites were found to be clustered (Figure 3D). Metabolites involved in tryptophan metabolism, such as quinoline-4,8-diol, kynurenic acid, and quinaldic acid, were significantly decreased in the candidemia group compared with the control group.

Spearman correlation analysis was used to identify possible relationships between altered gut microbiota composition and fecal co-metabolites. As shown in Figure 3E, linoleic acid metabolism, tryptophan metabolism, bile secretion, and arachidonic acid metabolism are associated with beneficial bacteria, such as *Lactococcus, Acidibacter, and Sphingomonas*. Thus, gut microbiota dysbiosis may be associated with fecal metabolites.

### The Characteristics of Kynurenic Acid

We screened three differentially expressed metabolites, quinoline-4,8-diol, kynurenic acid, and quinaldic acid (Table 1), and analyzed their contents in the peripheral blood of patients with invasive *C. albicans* infection and healthy controls by targeted mass spectrometry. There was a significant difference in the content of kynurenic acid in the peripheral blood of patients with invasive *C. albicans* infection compared with healthy controls. The production of kynurenic acid is closely related to *Clostridium sporogenes*, and its relative expression in the peripheral blood of patients with invasive *C. albicans* infection is significantly lower than other contents (*P <* 0.05) (Figure 1A). Further analysis of the physical and chemical characteristics using the HMDB metabolic database showed that kynurenic acid (HMDB0000715) is a kind of phosphatidic acid molecule with a molecular formula of C_10_H_7_NO_3_ and a molecular weight of 189.1675 Da (Figure 1B). Furthermore, the metabolic pathway of kynurenic acid was associated with the metabolism of tryptophan (Figure 1C).

**TABLE 1:** Tryptophan metabolites from intestinal flora.

Metabolites	VIP	*P*-value	log2(FC)	T1	T2	T3	T4	T5	T6	C1	C2	C3	C4	C5	C6
Quinoline-4,8-diol	4.352005841	0.000456894	−0.98202032	2578.587561	3520.021674	3697.555233	1843.168192	3303.395711	3228.180323	5454.841879	5656.129382	6014.92193	5524.328808	6625.031145	6616.462545
Kynurenic acid	3.310455392	5.6894E-05	−3.598873938	880.2494609	1046.944327	1066.181466	186.0590311	994.0790165	1041.443168	1772.814592	2128.751426	2904.104664	2858.509106	2939.756655	3192.521016
Quinaldic acid	1.860736485	1.26426E-05	−1.318803137	315.9288544	432.2407231	408.3470075	173.5258084	413.5516563	430.9509149	745.9731837	808.7144176	875.3788752	871.9824855	1027.467941	1095.082788

*T, Candida albicans infection group; C, Healthy control group (T vs. C).*

*Three differentially expressed metabolites were screened out: quinoline-4,8-diol, kynurenic acid, and quinaldic acid.*

### Kynurenic Acid Ameliorates Intestinal Injury Caused by Invasive *Candida albicans* Infection

**FIGURE 4:**
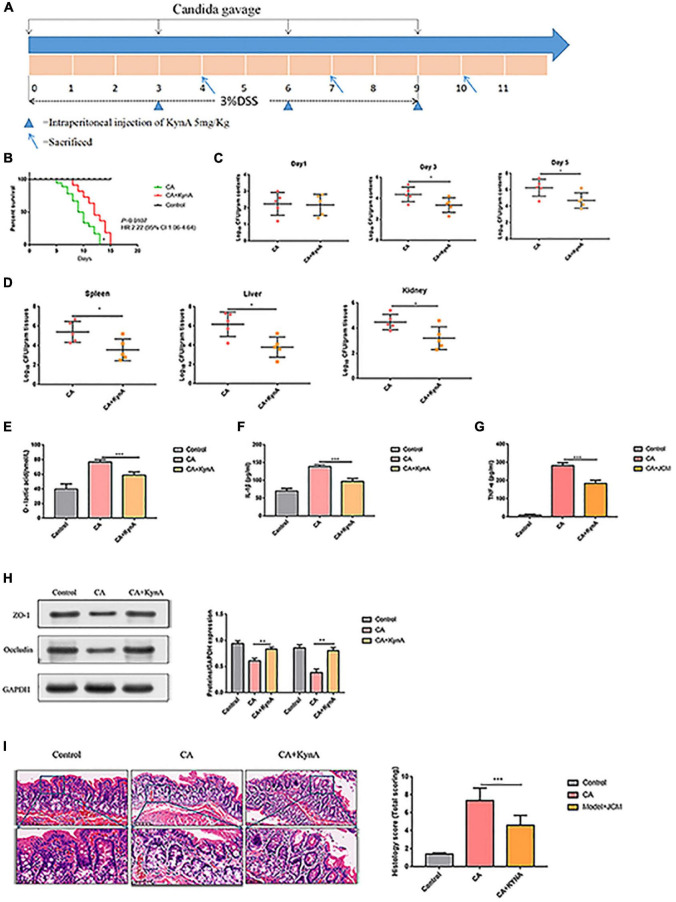
Kynurenic acid alleviates intestinal injury caused by invasive *C. albicans* infection. **(A)** Experimental design including KynA administration and Candida gavage. **(B)** Mice infected with *C. albicans* exhibited higher mortality than mice treated with KynA. **(C)** KynA treatment decreased the colonization of intestinal *C. albicans*. Fungal load in feces collected from untreated and KynA groups at 1, 3, and 5 days after infection. **(D)** Fungal load in kidneys, spleen, and liver samples from euthanized mice collected immediately in the KynA-treated groups and 10 days post-infection in the untreated groups. **(E–G)** The levels of D-lactic acid, IL-1β, and TNF-α were significantly decreased in the CA+KynA group compared to those of the CA group (*P* < 0.05). **(H)** Expression of ZO-1 and occludin in the intestinal epithelial cells of the CA+KynA group was significantly higher than in the CA group. **(I)** Chiu’s pathological scoring system revealed that mice from the CA+ KynA group had less intestinal mucosal damage than the mice in the CA group. Statistical significance was evaluated using the Mann–Whitney *U* test. *P*-values < 0.05 (*) or < 0.01 (**) were considered statistically significant. CA, *Candida albicans* infection; KynA, Kynurenic acid.

Mice infected with *C. albicans* experience higher mortality than mice treated with KynA [*P* = 0.01, HR 2.22 (95% CI: 1.06–4.64)], and no deaths occurred in the control group (Figure 4B). The colonization and proliferation of *C. albicans* in the intestinal tract of mice were measured quantitatively by culturing *C. albicans* in the feces of mice on days 4, 7, and 10 in both groups. On the first day after infection, there was no significant difference in the content of *C. albicans* between the groups. The *C. albicans* load in the CA+KynA group was significantly lower than that in the CA group on days 7 and 10 after infection (*P <* 0.01), suggesting that KynA can reduce the colonization of *C. albicans* in the intestinal tract (Figure 4C). On the fifth day after infection, the liver, kidney, and spleen were collected for analysis to determine the distribution and toxicity of *C. albicans* by fungal culture. The *C. albicans* load in the kidneys, spleen, and liver was significantly lower in the CA+KynA group than in the CA group (*P* < 0.05) (Figure 4D). The levels of D-lactic acid, IL-1β, and TNF-α were significantly decreased in the CA+KynA group compared to those of the CA group (*P* < 0.05) (Figures 4E–G). Zonula occludens-1 (ZO-1) and occludin are important integral membrane proteins that contribute to the structural integrity of tight junctions during the development of the intestinal mucosal barrier (Li et al., 2015). Compared to the CA group, the expression of ZO-1 and occludin proteins in the intestinal epithelial cells of mice from the CA+KynA group was significantly increased, indicating that KynA protected the intestinal barrier (Figure 4H, *P* < 0.01). The Chiu pathologic scores of mucosal injuries were used to assess the extent of the intestinal histological injury. HE results showed an intact colonic mucosa structure in the control group, closely arranged large intestine glands with no signs of ulcers on the epithelial cells, and more goblet cells than in the CA group. In contrast, there were ulcers in the superficial layer of the colonic mucosa in mice from the CA group, and the entire tissue structure of the mucosal layer was destroyed with signs of inflammatory cell infiltration. The impaired structure of the colonic mucosa was improved in the CA+KynA group, no obvious ulcers were observed, and epithelial cells were not significantly damaged, but the number of goblet cells was reduced. Chiu’s pathological scoring system revealed that mice from the CA+KynA group had less intestinal mucosal damage than the mice of the CA group (Figure 4I).

### Kynurenic Acid Activates Aryl Hydrocarbon Receptor Expression in the Intestinal Epithelium

**FIGURE 5:**
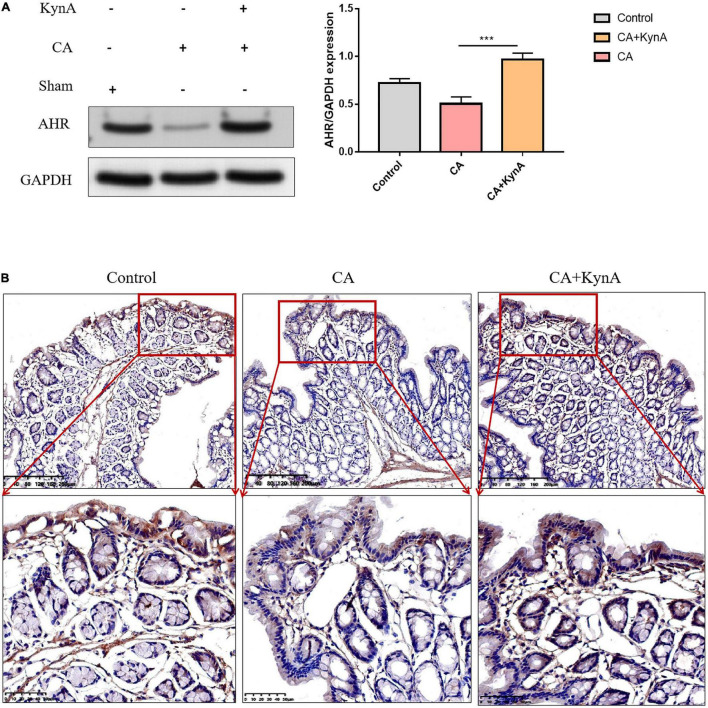
Kynurenic acid activates AHR expression in the intestinal epithelium. **(A,B)** Western blot analysis and immunohistochemistry show higher expression of AHR in the CA+KynA group than in the CA group. Statistical significance was evaluated using the Mann–Whitney *U* test. *P*-values < 0.05 (*) or < 0.01 (**) were considered statistically significant. CA, *Candida albicans* infection; KynA, Kynurenic acid.

We studied the effect of kynA on the expression levels of AHR in the intestinal epithelium. Western blot analysis showed that protein levels of AHR increased significantly in the intestinal epithelium of mice in the CA+KynA group compared to the mice of the CA group (*P <* 0.01). These results were confirmed by immunohistochemistry analysis (Figures 5A,B).

### KynA Suppressed the MLCK-pMLC Signaling Pathway by Activating Aryl Hydrocarbon Receptor

The increased expression of myosin light chain kinase (MLCK) is known to activate myosin light chain phosphorylation to induce contraction of the peri-junctional actomyosin ring, reducing intestinal permeability and improving the functions of the epithelial barrier (Xiong et al., 2016). To further investigate the effect of AHR activation on the MLCK-pMLC signaling pathway, we detected MLCK expression and MLC phosphorylation.

**FIGURE 6:**
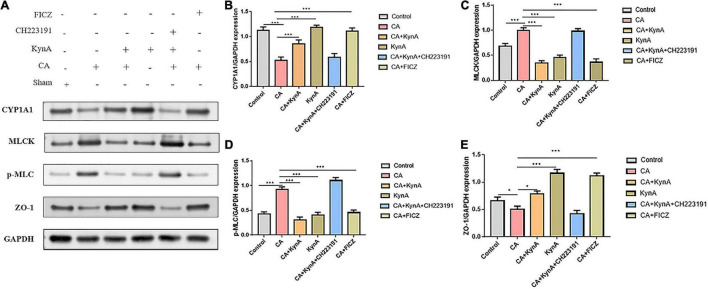
KynA activates the suppression of MLCK-pMLC signaling pathway by AHR. The detection of MLCK expression and MLC phosphorylation was carried out to determine the effect of AHR activation on the MLCK-pMLC signaling pathway. In Caco-2 cells, after KynA treatment, the expression of the AHR downstream protein CYP1A1 increased significantly, as did the expression of the tight junction proteins ZO-1 and occludin. The expression of MLCK and pMLC decreased significantly. In the CA-Caco-2 cell model, expression levels of CYP1A1, ZO-1, and occludin were significantly decreased, but the expression levels of MLCK and pMLC increased. After treatment with KynA and the AHR agonist FICZ, expression of CYP1A1 increased significantly, as did the expression of ZO-1 and occludin, but the expression of MLCK and pMLC decreased. Treatment with an AHR inhibitor (CH223191), led to a decrease in the expression of CYP1A1, ZO-1, and occludin, and to an increase in the expression of MLCK and pMLC. Statistical significance was evaluated using the Mann–Whitney *U* test. *P*-values < 0.05 (*) or < 0.001 (***) were considered statistically significant. CA, Candida albicans infection; KynA, Kynurenic acid; FICZ, 6-Formylindolo[3,2-b]carbazole.

In Caco-2 cells, after treatment with KynA alone, the expression of the AHR downstream protein CYP1A1 increased significantly, as did the expression of the tight junction proteins ZO-1 and occludin. However, the expression of MLCK and pMLC decreased significantly (*P <* 0.01). The expression levels of CYP1A1, ZO-1, and occludin proteins were significantly reduced in the CA-Caco-2 cell model, whereas the levels of MLCK and pMLC were significantly higher (*P <* 0.01). After treatment with KynA and the AHR agonist FICZ, the expression of CYP1A1 in Caco-2 cells increased significantly, as did the expression of the tight junction proteins ZO-1 and Occludin (*P <* 0.01), whereas the expression of MLCK and pMLC decreased significantly (*P <* 0.01). However, after treatment with an AHR inhibitor (CH223191), the expression of CYP1A1, ZO-1, and occludin proteins decreased significantly, whereas the expression of MLCK and pMLC increased (*P <* 0.01). These findings suggest that AHR activation might protect the intestinal epithelial barrier from disruption caused by *C. albicans* infection by suppressing the MLCK-pMLC signaling pathway. Therefore, we suggest that KynA can inhibit the MLCK-PMLC signaling pathway by activating the AHR receptor and promoting the expression of intestinal tight junction proteins, thereby helping to maintain the integrity of the intestinal barrier (Figure 6).

### KynA Suppressed the MK2-P-MK2 Signaling Pathway by Activating Aryl Hydrocarbon Receptor

The P38/MK2 [mitogen-activated protein kinase (MAPK)-activated protein kinase-2, also known as MAKAP kinase-2] is a member of the mitogen-activated protein kinase (MAPK) family with a role in inflammation (Newton and Holden, 2006). The p38-MAPK/MK2 signaling pathway leads to tristetraprolin (TTP) phosphorylation, resulting in its proteasomal degradation (Huang et al., 2016). Several reports have shown that AHR activation can have an anti-inflammatory effect *in vitro* and *in vivo* during inflammatory processes and immune responses (Brandstatter et al., 2016). However, the effect of AHR activation on MK2 has not been reported (Riemschneider et al., 2021). Therefore, we investigated the regulation of TTP and changes in the expression of MK2 and p-MK2 following AHR activation at the cellular level.

**FIGURE 7:**
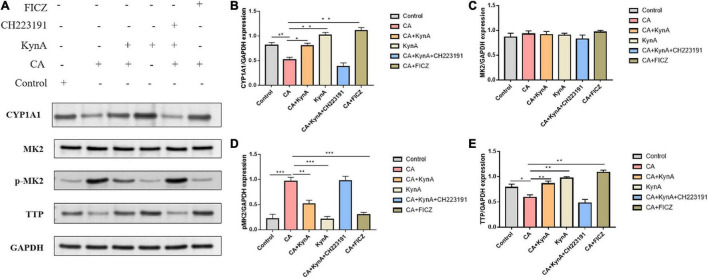
KynA suppressed the MK2/p-MK2 signaling pathway by activating AHR. We investigated the regulation of TTP and expression changes of MK2 and P-MK2 induced by AHR activation at the cellular level. In Caco-2 cells, after treatment with KynA, expression of CYP1A1 increased significantly, as did expression of TTP also (*P <* 0.05), while expression of MK2 decreased significantly (*P <* 0.05). In the CA- Caco-2 cell model, the expression of proteins CYP1A1 and TTP decreased significantly, but the expression of p-MK2 increased (*P <* 0.01). After the treatment of Caco-2 cells with KynA and the AHR agonist FICZ, the expression of CYP1A1 and TTP was significantly increased, but the expression of p-MK2 decreased significantly (*P <* 0.01). Treatment with an AHR inhibitor (CH223191) led to a decrease in the expression of CYP1A1 and TTP, and an increase in the expression of p-MK2 (*P <* 0.01). Statistical significance was evaluated using the Mann–Whitney *U* test. *P*-values < 0.05 (*) or < 0.01 (**) were considered statistically significant. CA, *Candida albicans* infection; KynA, Kynurenic acid; FICZ, 6-Formylindolo[3,2-b]carbazole. *P*-values < 0.001 (***).

In Caco-2 cells, KynA treatment led to significant increases in the expression levels of CYP1A1 and TTP (*P <* 0.05) but to a significant decrease in MK2 expression (*P <* 0.05). Following infection of Caco-2 cells with *C. albicans*, the expression levels of proteins CYP1A1 and TTP decreased significantly, but the expression levels of p-MK2 increased (*P <* 0.01). FICZ, a KynA and AHR agonist, significantly increased the expression levels of CYP1A1 and TTP in Caco-2 cells but decreased p-MK2 (*P <* 0.01). However, after treatment with the AHR inhibitor CH223191, the expression of CYP1A1, TTP, and p-MK2 was similar to that in the CA group (*P <* 0.01). No significant differences were found in the expression levels of MK2. These findings suggest that AHR regulates TTP expression *via* the MK2/p-MK2 pathway. Our study demonstrates that KynA increased the expression of TTP by activating the AHR receptor, which further suppressed the activation of the MK2/p-MK2 pathway (Figure 7).

## Discussion

*Candida* is one of the most common fungi of the GI tract; however, bacterial dysbiosis can cause *Candida* commensalism to become pathogenic, resulting in prolonged infections and *Candidiasis* (Strati et al., 2016). Sepsis can lead to the impairment of the intestinal barrier structure and function due to inflammation, ischemia, and hypoxia (Quan et al., 2020). Invasive *C. albicans* infection can further impair the intestinal barrier and lead to disseminated candidiasis, which disrupts the microflora of the gut (Geng et al., 2020). The extent of inflammation is correlated to the severity of gut microbiota dysbiosis (Lin et al., 2019).

In this study, we examined the alterations in intestinal flora in patients with candidemia compared to the intestinal flora of healthy individuals. At the genus level, the relative abundances of *Lachnoclostridium*, *Bacteroides*, and *Lactococcus* were significantly decreased in patients with candidemia compared to healthy individuals. Metabolites involved in tryptophan metabolism, such as quinoline-4,8-diol, kynurenic acid, and quinaldic acid, were also significantly decreased in these patients. We investigated a possible relationship between altered gut microbiota composition and fecal co-metabolites by Spearman correlation analysis, and showed that gut microbiota dysbiosis is closely associated with intestinal metabolites. Metabolites derived from gut microbiota can regulate host immune function and are involved in the metabolic functions of the host. One example of such an organism is *L. reuteri*, which prevents gastrointestinal disturbances, such as diarrhea, by restoring microbial flora and regulating intestinal immune function (Wang et al., 2020). Recent studies in gut microbial metabolomics have demonstrated that an increased abundance of probiotics could increase the concentration of bacterial metabolites, such as propionate and butyrate, thus enhancing the activity of immune cells in the gut (Xu et al., 2020). The production of kynurenic acid is significantly lower in patients with invasive *C. albicans* infection. By using an established mouse model of disseminated candidiasis, we showed that KynA treatment could alleviate the intestinal inflammatory response and decrease the production of serum inflammatory mediators, thus preserving the intestinal barrier function. Therefore, our results suggest a protective role for the intestinal metabolism in intestinal barrier maintenance.

Aryl hydrocarbon receptor is a ligand-dependent transcriptional factor that is widely expressed in barrier tissues consisting of immune cells, epithelial cells, endothelial cells, and stromal cells (Zhou et al., 2019). A number of studies have shown that AHR and AHR agonists maintain gut health and protect against intestinal diseases (Jin et al., 2014). AHR activation in the intestinal tract is dependent on the concentration of tryptophan and its metabolites (Wei et al., 2018). Kynurenine is synthesized from tryptophan (Williams et al., 2014), which has been shown to activate AHR (Moffett et al., 2020). In this study, we found that KynA activated AHR receptors in the intestine both *in vitro* and *in vivo*. Recent research suggested that AHR activation might help preserve the intestinal epithelial barrier function and protect it from disruption caused by TNF-alpha/IFN-gamma, by suppressing the MLCK-pMLC signaling pathway (Yu et al., 2018).

We used Caco-2 cells co-cultured with *C. albicans* to verify the effect of AHR activation on cell damage caused by infection and the role of the MLCK-pMLC signaling pathway. Our findings showed that KynA inhibited the MLCK-pMLC signaling pathway by activating the AHR receptor, promoting the expression of intestinal tight junction proteins, and helping in maintaining the intestinal barrier intact. TTP is a p38-MAPK/MK2 kinase target that changes its RNA affinity when phosphorylated (Perlewitz et al., 2010). In the absence of AHR, disruption of the intestinal barrier was increased in the colitis model, whereas FICZ activated AHR to alleviate DSS-induced colitis *via* the MK2/p-MK2/TTP pathway (Wang Q. et al., 2018). KynA increased TTP expression by activating the AHR receptor, which subsequently suppressed the activation of the MK2/p-MK2 pathway.

In conclusion, this study demonstrated that the intestinal metabolite KynA can protect against intestinal injury induced by C. albicans by activating AHR involved in regulating the MLCK-pMLC signaling pathway and MK2-p-MK2 signaling pathway, which would offer new potential strategies for the clinical treatment of invasive *C. albicans* infection.

## Data Availability Statement

The datasets presented in this study can be found in online repositories. The names of the repository/repositories and accession number(s) can be found below: https://www.ncbi.nlm.nih.gov/, PRJNA835672.

## Ethics Statement

The studies involving human participants were reviewed and approved by the Shanghai Fifth People’s Hospital, Shanghai, China. The patients/participants provided their written informed consent to participate in this study. The animal study was reviewed and approved by the East China Normal University’s Animal Center.

## Author Contributions

All authors listed have made a substantial, direct, and intellectual contribution to the work, and approved it for publication.

## Conflict of Interest

The authors declare that the research was conducted in the absence of any commercial or financial relationships that could be construed as a potential conflict of interest.

## Publisher’s Note

All claims expressed in this article are solely those of the authors and do not necessarily represent those of their affiliated organizations, or those of the publisher, the editors and the reviewers. Any product that may be evaluated in this article, or claim that may be made by its manufacturer, is not guaranteed or endorsed by the publisher.
